# Supplementary material for: Effect of Atomoxetine on Behavioral Difficulties and Growth Development of Primary School Children with Attention-Deficit/Hyperactivity Disorder: A Prospective Study
Source: Children (Basel). 2022 Feb 6;9(2):212. doi: 10.3390/children9020212 (PMC8870549; doi:10.3390/children9020212)
Supplement: Supplementary file 1 [file children-09-00212-s001.zip › children-1564260-supplementary.pdf]

Detailed information of serum IGF-1 and IGFBP-3 levels at baseline

IGF-1 (ng/ml)

Age (years)	Normal Range*	Girl (N=56)			Boy (N=93)		
		mean	SD	N	mean	SD	N
6-7	64.31~312.27/ girl<br>52.71~354.71/ boy	172.71	44.75	7	181.67	35.63	9
7-8	68.62~352.18/ girl<br>52.29~419.85/ boy	150.88	27.89	8	184.94	44.50	17
8-9	110.08~413.24/ girl<br>108.38~444.66/ boy	166.10	34.27	15	169.17	30.04	28
9-10	83.58~495.34/ girl<br>83.84~359.96/ boy	169.00	27.95	16	166.25	29.02	22
10-11	100.01~691.17/ girl<br>122.23~525.23/ boy	143.50	14.62	4	162.67	32.73	9
11-12	284.32~857.12/ girl<br>174.61~609.89/ boy	175.50	32.35	6	183.38	18.88	8

IGFBP-3 (μg/ml)

Age (years)	Normal Range*	Girl (N=56)			Boy (N=93)		
		mean	SD	N	mean	SD	N
6-7	2.39~11.19/ girl<br>2.65~7.89/ boy	4.21	0.40	7	4.28	0.46	9
7-8	3.49~11.67/ girl<br>3.34~7.66/ boy	4.52	0.51	8	4.21	0.60	17
8-9	4.06~11.58/ girl<br>3.35~8.15/ boy	4.28	0.65	15	4.08	0.48	28
9-10	3.39~11.83/ girl<br>2.32~7.40/ boy	4.25	0.35	16	4.21	0.39	22
10-11	4.33~12.25/ girl<br>2.63~8.43/ boy	3.84	0.26	4	3.95	0.35	9
11-12	6.60~13.80/ girl<br>2.84~8.48/ boy	4.26	0.73	6	4.17	0.51	8

*** The normal range of IGF-1 and IGFBP-3 levels were based on the literature[1]**

1. Xu, S., et al., *A study of normal reference values for serum insulin growth factor-1 and insulin factor-binding protein-3 in children and adolescents.* Journal of Clinical Pediatrics, 2009. **27**(12): p. 1105-1110.
